# Palliative Care Physicians' Religious / World View and Attitude Towards Euthanasia: A Quantitative Study Among Flemish Palliative Care Physicians

**DOI:** 10.4103/0973-1075.53511

**Published:** 2009

**Authors:** B Broeckaert, J Gielen, T Van Iersel, S Van den Branden

**Affiliations:** Inter-disciplinary Centre for the Study of Religion and Worldview, K.U. Leuven, Belgium

**Keywords:** Euthanasia, Palliative Care, Religion

## Abstract

**Aims:**

To Study the religious and ideological views and practice of Palliative Care physician towards Euthanasia.

**Materials and Methods:**

An anonymous self administered questionnaire approved by Flemish Palliative Care Federation and its ethics steering group was sent to all physicians(n-147) working in Flemish Palliative Care. Questionnaire consisted of three parts. In first part responded were requested to provide demographic information. In second part the respondents were asked to provide information concerning their religion or world view through several questions enquiring after religious or ideological affiliation, religious or ideological self-definition, view on life after death, image of God, spirituality, importance of rituals in their life, religious practice, and importance of religion in life. The third part consisted of a list of attitudinal statements regarding different treatment decisions in advanced disease on which the respondents had to give their opinion using a five-point Likert scale.99 physician responded.

**Results:**

We were able to distinguish four clusters: Church-going physicians, infrequently church-going physicians, atheists and doubters. We found that like the Belgian general public, many Flemish palliative care physicians concoct their own religious or ideological identity and feel free to drift away from traditional religious and ideological authorities.

**Conclusions:**

In our research we noted that physicians who have a strong belief in God and express their faith through participation in prayer and rituals, tend to be more critical toward euthanasia. Physicians who deny the existence of a transcendent power and hardly attend religious services are more likely to approve of euthanasia even in the case of minors or demented patients. In this way this study confirms the influence of religion and world view on attitudes toward euthanasia.

## INTRODUCTION

For many centuries Flanders (Belgium) has been a region where religion meant Christianity and Christianity meant Catholicism. However, in the course of the last four decades, church attendance in West-European countries like Belgium has declined tremendously.[1] In the same period, palliative care programmes have been developed in these countries and palliative care has gradually gained public support. The simultaneous decline in church attendance and the growth of Palliative Care services in Western Europe may seem remarkable as the founders of the modern palliative care movement clearly drew inspiration from religion in general and Christianity in particular.[2] If religion was so important in the early years of the modern hospice movement, how could palliative care thrive in times in which declining church attendance seems to imply a decrease in interest in religion? One possible solution for this paradox is the hypothesis of the secularization of the hospice movement. According to this theory religious concerns of palliative care have been rethought in a secular manner and the medical, psychosocial, and educational aspects of palliative care have become more prominent.[3] Thus, palliative care no longer needs a religious society to flourish. Recent surveys among the general public point toward another possible explanation for this paradox. These surveys show that religion and, more broadly speaking, world view is a multifaceted phenomenon that encompasses much more than just the attendance of religious services. In Western Europe many people have bid the church farewell, yet remain interested in religious issues.[1,4] If a similar attitude toward religion was found among Western European palliative care physicians, religion could still spark physicians' willingness to devote their efforts to the care of the terminally ill.

On the basis of the present state of research, it is impossible to judge whether the religious or ideological views of Western European palliative care physicians differ from other physicians or from the general public. So far, no survey has been conducted among a group of Western European palliative care physicians assessing their religious or ideological practice as well as their views on a broad range of religious and ideological issues. To a certain extent, the religion or world view of European palliative care physicians is analyzed in surveys assessing the influence of religion on palliative care physicians' attitudes toward certain treatment decisions in advanced disease.[5–8] Unfortunately in these studies the enquiry after religious and ideological views is restricted to one question, and hence fails to accurately capture the complexity of the phenomenon religion and world view. In 1995 Cornette undertook a quantitative survey among palliative care nurses, physicians, pastors, counsellors and volunteers about their opinions regarding spiritual care. Since an assessment of the religious and ideological views and practices of the palliative care providers was not the prime focus of the study, the enquiry in this regard was restricted to prayer, mass attendance, belief in a personal God and life after death.[9,10]

Outside Europe more specific research has been undertaken regarding palliative care physicians and spirituality.[11,12] Yet, spirituality is a very broad concept that also includes general existential issues.[13,14] As a consequence, these studies pay little attention to the specific religious or ideological views and practices of the physicians. Moreover, in these studies, the number of physicians being surveyed is limited. It would, however, be highly interesting to know more about the religious or ideological views of palliative care physicians since such knowledge could enable us to understand why palliative care physicians are willing to work in palliative care and why they judge certain ethical decisions (e.g. palliative sedation; euthanasia) to be acceptable or not.

Therefore, we intended to study the religious and ideological views and practices of palliative care physicians. Most studies which attempt to measure the religious and ideological views and practices of physicians restrict their enquiry to the religious or ideological affiliation of the physicians. This is also the case in surveys assessing the relationship between religion and attitudes toward euthanasia.[8,15–19] Religious or ideological affiliation is indeed helpful to understand the religious or ideological background of the respondents. Yet, as such, religious or ideological affiliation reveals little about how a respondent interprets his or her affiliation, and whether he or she diverts from the standard views and practices of his or her religious community. For that reason, we wanted to divide the physicians into different groups, with physicians giving similar answers on divergent religious or ideological questions.

In 2006, the Interdisciplinary Centre for the Study of Religion and World View (K.U.Leuven) and the Flemish Palliative Care Federation decided to undertake a quantitative study of the religious and ideological views and practices of Flemish palliative care physicians and their attitudes toward different treatment decisions in advanced disease. For the Interdisciplinary Centre for the Study of Religion and World View this survey is part of a series of qualitative and quantitative empirical studies, analyzing attitudes toward treatment decisions in advanced disease and the influence of religion and world view on these attitudes. Specific focus is being put on the influence of Islam, Judaism, Hinduism and Christianity. This article deals with the religious and ideological views and practices of Flemish palliative care physicians and more specifically with the question how these physicians could be classified regarding their religion or world view and how their religion or wordview influences their attitudes toward euthanasia.

## MATERIALS AND METHODS

In the middle of May 2006 an anonymous self-administered questionnaire was sent to all physicians (*n* = 147) working in Flemish palliative care. The addresses of the physicians had been provided by the Flemish Palliative Care Federation. Earlier, the questionnaire had been presented for evaluation to a team of palliative care experts and sociologists. The questionnaire had also been approved by the Flemish Palliative Care Federation and its ethics steering group. The questionnaire (in Dutch) consisted of three parts. In the first part, the respondents were requested to provide demographic information, including gender, age and years of experience in palliative care. In the second part, the respondents were asked to provide information concerning their religion or world view through several questions enquiring after religious or ideological affiliation, religious or ideological self-definition, view on life after death, image of God, spirituality, importance of rituals in their life, religious practice, and importance of religion in life. This part of the questionnaire has been translated into English and added as an appendix to the article_*_. The accuracy of this translation was verified through the method of back translation with the help of a native English speaker. The third part consisted of a list of attitudinal statements regarding different treatment decisions in advanced disease on which the respondents had to give their opinion using a five-point Likert scale. Approximately, 30 to 40 minutes were needed to complete the questionnaire. Together with the questionnaires the physicians received stamped return envelopes to mail back completed questionnaires. The physicians also received a stamped card on which they were requested to write their name. The card had to be sent separately to the researchers after posting the completed questionnaire to avoid follow-up mailings. In the beginning of June a follow-up mailing reminding the physicians about the questionnaires, was sent to all physicians whose response card had not been received. At the end of June a third mailing was sent again to all physicians whose response card had not been received by then. The third mailing contained a new questionnaire, stamped return envelope, response card, and a letter reminding them about the questionnaire and kindly requesting them to complete and post the questionnaires. The cut-off date for responses was 31 August, 2006; 99 physicians responded (67.3%). For the statistical analysis of the data R 2.4.1 was used (www.r-project.org).

## RESULTS

There were 64 male physicians (64.6% of all physicians, one no response). The mean age was 46.1 (std. dev. 9.4). The mean amount of years of professional experience in palliative care was 7 (std. dev. 3.9, five no response).

### Religious or ideological affiliation and self-definition

Following the questionnaire of the European Value Study, we made a distinction between religious or ideological affiliation and religious or ideological self-definition.[1] In the item relating to religious or ideological self-definition (item 1 of the questionnaire in the appendix) the respondents could choose from a list which term best describes their own conviction regarding their personal view on religion or world view. While 33 physicians chose Christian, 22 Catholic, one evangelical, eight Atheist, nine Agnostic, six free-thinker, one other world view or religion, four- none of the aforementioned possibilities, because I myself determine what I believe, and two - I am indifferent toward world view and religion, Protestant, Other Christian denomination, Jewish, and New Age were also mentioned as options on the list. No respondents solely chose any of these possibilities. In the numbers given above we did not include, respondents who indicated two answers. Five respondents chose Christian and Catholic. The following combinations were made each by one physician: Christian and Agnostic, Christian and Buddhist, Christian and New Age, Catholic and other world view or religion, Atheist and None of the aforementioned possibilities, because I myself determine what I believe, Atheist and Free-thinker, Christian and None of the aforementioned possibilities, because I myself determine what I believe.

In the item relating to religious or ideological affiliation (item 4) the respondents could indicate whether they consider themselves a member of a religious or ideological community and which community that is. About 57 physicians chose Catholic Church, two preferred-Other Christian community, three chose the Free-thinking community, and 28 opted for - I do not belong to any group. Jewish community, Protestant Church (United Protestant Churches of Belgium), Evangelical Church, Islamic community, Buddhist community, and Others were mentioned as options on the list. No respondents solely indicated any of these answers. In the numbers, respondents who chose two answers have not been included. All but one of the four respondents mentioned two communities, combined “Catholic Church” with another possibility (resp. “Other Christian community”, “Other”, and “Evangelical Church”). One physician chose “Other” and “I do not belong to any group”.

### Religious and ideological clusters

**Figure 1:**
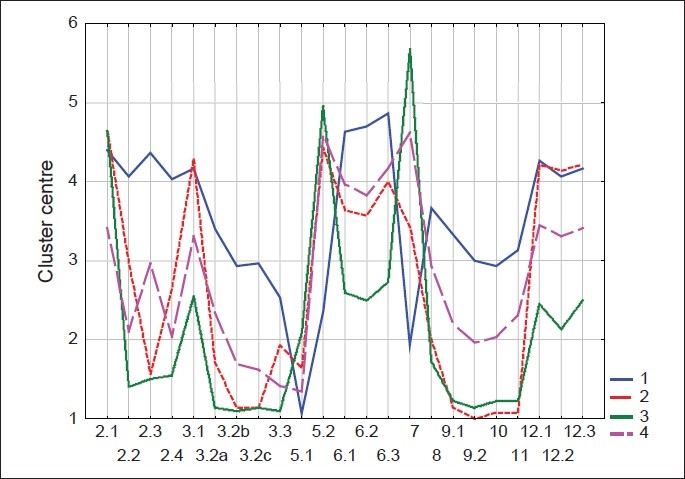
Religious and ideological cluster centre

We attempted to get a more accurate picture of the respondents' religious and ideological views and practices through a list of questions or statements regarding religious and ideological issues (cf. appendix). The respondents were requested to express their opinions or formulate an answer by choosing one answer from the answer categories. The items in the questionnaire dealt with many diverging aspects of religion and world view. Our main aim, however, was not to measure particular religious or ideological views and practices, but rather to obtain an overall picture of the nurses' religion and world view. For this purpose, we performed a traditional cluster algorithm on the answers on the different questions dealing with religion and world view. The items about religious or ideological self-definition (item 1) and religious or ideological affiliation (item 4) were not included in the cluster analysis, since these items could not be interpreted as continuous data. We recoded items 8 and 10, which dealt with image of God and life after death respectively. In item 8, the first three answer categories were left unaltered, while the last five categories were taken together, thus forming answer category four, meaning “a specific image of transcending reality”. In this way, the answer categories were ordered gradually, from unbelieving over vague image of the transcending reality to specific image. A similar procedure was followed for item 10. Also, here, the first three answer categories were not changed. The last four answer categories were combined as fourth answer category, implying “determined image of life after death”. Four clusters were retained. The cluster centres were plotted to enable us to label each cluster [Figure 1]. The numbers on the horizontal axis refer to the different items of the questionnaire as found in the appendix.

### Cluster centres

30 physicians (31.6% of all physicians whose cluster membership could be determined) belong to the first cluster. The average physician in this group believes in a reality that transcends human existence. Often these physicians have a clear idea about what this transcending reality looks like. They also believe in life after death. They are active church members: They find rituals important, often attend religious services and pray regularly. They find religion and world view important for their personal and professional life. We called physicians belonging to this cluster ‘church-going respondents’.

In the second cluster 14 physicians (14.7%) are found. Physicians in this cluster hardly attend religious services and do not find rituals important, except on feast days and at the important moments of life. Generally they do not know whether a transcendent reality exists and they do not believe in life after death. Members of this cluster were called ‘infrequently church-going respondents’.

The third cluster groups 22 physicians (23.2%) who are clearly atheist. The obvious name for physicians belonging to this cluster is ‘atheists’. Most respondents in this group do not believe in a transcendent reality, do not pray, do not attend religious services and find rituals not essential even at important moments of life.

There are 29 physicians (30.5%) in the fourth cluster. Physicians of this category have serious doubts about faith and belief. They doubt about the existence of a transcendent reality. They do not know if there will be life after death or what that life may be like. They seldom pray, and they find rituals not very necessary except at important moments of life. The label we gave to this cluster is ‘doubters’.

When we check whether there are differences between the clusters on the level of the demographic variables gender, age, and years of experience in palliative care, no significant differences (α = 0.05) are found [Table 1]

**Table 1:** Effect of demographic variables on clusters

	Cluster 1 (n = 30) Church-going respondents	Cluster 2 (n = 14) Infrequently church-going respondents	Cluster 3 (n = 22) Atheists	Cluster 4 (n = 29) Doubters	Test statistic
Gender					$x_{3}^{2}=2$, *P* = 0.6^1^
Male	72% (21)	64% (9)	55% (12)	66% (19)	
Female	28% (8)	36% (5)	45% (10)	34% (10)	
Age	41 49 57 (48 ± 11)	40 48 52 (47 ± 7)	38 42 48 (43 ± 7)	37 48 50 (45 ± 9)	F_3,89_ = 1, *P* = 0.3^2^
Years of experience	4 6 12 (8 ± 5)	5 7 8 (7 ± 3)	5 6 10 (7 ± 3)	4 6 10 (7 ± 4)	F_3,86_ = 0.2, *P* = 0.9^2^

*a b c* represent the lower quartile *a*, the median *b*, and the upper quartile *c* for continuous variables. x ± s represents X ± 1 SD. Numbers after percents are frequencies. Tests used:

1 Pearson test

2 Kruskal-Wallis test

We also checked if there were differences between the clusters on the level of religious or ideological self-definition [Table 2] and religious or ideological affiliation [Table 3].

**Table 2:** Religious or ideological self-definition

Which term best describes your own conviction (regarding your personal view on religion or world view)?	*N*	Cluster 1 Church-going respondents	Cluster 2 Infrequently church going respondents	Cluster 3 Atheists	Cluster 4 Doubters
Christian	33	9	4	2	16
Catholic	22	13	0	3	5
Protestant	0	0	0	0	0
Evangelical	1	1	0	0	0
Other Christian denomination	0	0	0	0	0
Atheist (I do not believe in god or gods)	8	0	2	6	0
Agnostic (I let in different possibilities)	9	0	3	3	2
Free-thinker	6	0	2	2	2
New age	0	0	0	0	0
Other world view or religion	1	0	1	0	0
None of the aforementioned possibilities, because I myself determine what I believe	4	1	0	2	1
I am indifferent toward world view and religion	2	0	0	2	0
Christian and Catholic	5	3	1	0	1
Other combination	7	2	1	2	2

One physician did not answer this question

**Table 3:** Religious or ideological affiliation

Do you consider yourself a member of a religious or ideological community?	N	Cluster 1 church-going respondents	Cluster 2 infrequently church-going respondents	Cluster 3 atheists	Cluster 4 doubters
Catholic church	57	26	5	8	17
Protestant church (United Protestant Churches of Belgium)	0	0	0	0	0
Evangelical church	0	0	0	0	0
Other Christian community	2	0	0	1	0
Islamic community	0	0	0	0	0
Jewish community	0	0	0	0	0
Buddhist community	0	0	0	0	0
Free-thinking community	3	0	2	1	0
Other	0	0	0	0	0
I do not belong to any group	28	1	5	11	10
More than one community	4	0	4	0	0

A majority of the church-going respondents chose “Catholic” as religious or ideological self-definition. Nevertheless, many of the church-going respondents preferred the more neutral term “Christian”. Among the infrequently church-going respondents (cluster 2) and the doubters (cluster 4) the term “Christian” was more popular than “Catholic”. Among the cluster of the atheists (cluster 3) “Atheist” was the most frequently chosen option, though remarkably even in this cluster five respondents defined themselves as “Christian” or “Catholic” [Table 4].

**Table 4:** Comparison between religious or ideological clusters and euthanasia clusters

Religioideological clusters	n	Cluster1 (n = 45) Staunch advocates	Cluster 2 (n = 11) Opponents	Cluster 3 (n = 32) Intermediate group
Cluster 1:Church-going respondents	26	6 (13.3, 23.1)	8 (72.3, 30.8)	12 (37.5, 46.2)
Cluster 2:Infrequently church-going respondents	13	10 (22.2, 76.9)	1 (9.1, 7.7)	2 (6.2, 15.4)
Cluster 3:Atheists	21	14 (31.1, 66.7)	0	7 (21.9, 33.3)
Cluster 4:Doubters	28	15 (33.3, 53.6)	2 (18.2, 7.1)	11 (34.4, 39.3)

a (b, c) represent the number of physicians that belongs to a particular religious or ideological cluster and the respective euthanasia cluster (a), the % of physicians of the relevant euthanasia clusters that belongs to a particular religious or ideological cluster (b), the per cent of physicians of the relevant religious or ideological cluster that belongs to a particular euthanasia cluster

A large majority of the church-going respondents (cluster 1) and doubters (cluster 4) mentions “Catholic Church” as the religious or ideological community they belong to. Among the infrequently church-going respondents (cluster 2) and, rather surprisingly, the atheists (cluster 3) too a significant minority attests to be a member of the Catholic Church. This dominance of the Catholic Church is confirmed by the answers on the questions asking what kind of religious or ideological services the respondent attends now or used to attend when he or she was ten years old (item 5). While 93 respondents (96.9%) attended Catholic services when they were ten years old, when asked which religious or ideological services the respondents attended now, 54 respondents (96.4% of those respondents who answered this question) stated they attended Catholic services; 43 (43.4%) did not answer this question, most likely because they hardly or never attend religious or ideological services and were asked not to answer this additional question.

In another part of our study, to be published elsewhere, we studied the attitudes of the Flemish palliative care physicians toward euthanasia. In that part too we were able to distinguish a number of clusters. The Table below shows how the physicians are divided over both the different religious or ideological clusters and the euthanasia clusters, thus giving us an idea of the relationship between religion and worldview on the one hand and attitudes toward euthanasia on the other.

When compared to the other religious or ideological clusters, the church-going respondents much more frequently belong to the opponents of euthanasia. The cluster of the church-going respondents is the only cluster with a significant amount of its members also being part of the opponents of euthanasia. The church-going respondents have also the strongest presence in the intermediate euthanasia cluster, though it must be noted that six of the 26 physicians in this group are staunch advocates of euthanasia. On the other hand, a large majority of the atheists belongs to the cluster of the staunch advocates of euthanasia. None of the atheists can be characterised as an opponent of euthanasia. The infrequently church-going respondents and religious or ideological doubters are most often staunch advocates of euthanasia. Most religious or ideological doubters are staunch advocates of euthanasia.

In order to find out if there is a statistically significant association between the religious or ideological clusters and the euthanasia clusters, we performed a Pearson Chi-squared test. The association is highly significant ($x_{3}^{2}$ = 19.5, *P* = 0.003). To assess if the association remains significant when the covariates gender, age and years of experience in palliative care are taken into consideration, the multinomial logit model was used. A likelihood ratio test was performed. The likelihood ratio test is clearly significant (deviance full model 139.96, deviance reduced model 162.04, *P* = 0.005). There is a significant effect of the religious or ideological clusters on the euthanasia clusters also when the results are adjusted for the covariates gender, age and years of experience in palliative care.

## DISCUSSION

As a group, Flemish palliative care physicians cannot be called irreligious. Only 23.2% is distinctly atheist (cluster 3). Strong traditional believers make up for 31.6% of all physicians (cluster 1). In between these two extremes, we find the two remaining clusters (2 and 4) together comprising 44.7% of all physicians. Following the terminology of Dobbelaere and Voyé, the religious and ideological views and practices of physicians belonging to these two clusters could be described with the nouns “ambivalence” and “estrangement”.[1] These physicians still feel somehow connected with the Christian and more specific Catholic tradition. Nevertheless, they do not faithfully follow all traditional religious practices, convictions and beliefs. Our comparison of the religious and ideological clusters with the religious or ideological self-definition and affiliation further shows that also among physicians belonging to the cluster of the church-going respondents and the cluster of the atheists there is “ambivalence” and “estrangement”. In the cluster of the atheists several respondents chose “Christian” or “Catholic” as religious or ideological self-definition, and “Catholic Church” as religious or ideological affiliation. Likewise, some members of the cluster of the church-going respondents described their own religion or world view as Christian, while all but one member of the same cluster indicated “Catholic Church” as religious or ideological affiliation. Dobbelaere and Voyé explained the distinction between Catholic and Christian, as an expression of dissociation from the institutional Church. Those respondents who feel discontented with ecclesiastical teachings would be more inclined to opt for “Christian” rather than “Catholic”.

We did not observe a significant effect of any of the demographic variables (age, gender, and years of experience in palliative care) on the clusters. The absence of any relationship is remarkable since often women are said to be more religious than men, and older people are assumed to be more religious than younger people.[1,4,20] In an earlier study about the religious views of a sample of Belgians, Dobbelaere and Voyé modified the theory that women are more religious than men, by making a distinction between women who work outside the house and those women who do not. Dobbelaere and Voyé observed that the religious views and practices of women who work outside the house are closer to the practices and views of male respondents.[20] The distinction between women working outside the house or not may explain the lack of a relationship between gender and religion or world view in our survey, as all our respondents, both men and women, work outside the house.

Earlier European and Belgian surveys showed that older respondents are more religious than younger respondents.[1,4] In our survey we did not observe such difference. Maybe the differences in age between our respondents were too limited to show an influence of age on religious and ideological views and practices.

Earlier surveys, investigating the influence of religion on the attitudes of physicians toward euthanasia, observed that religiosity negatively influences approval of euthanasia.[17] In our research we noted that physicians who have a strong belief in God and express their faith through participation in prayer and rituals, tend to be more critical toward euthanasia, though in our study only a minority (30.8%) of these respondents could be called an opponent of euthanasia. Physicians who deny the existence of a transcendent power and who hardly attend religious services, are more likely to approve of euthanasia even in ethically difficult cases, like the cases of minors or demented patients. In our survey we found no atheist respondent that could be termed an opponent of euthanasia. It is clear from our study that religion and worldview is an important factor determining attitudes toward euthanasia, taking into account that the influence of atheism is probably more unequivocal than that of religion.

## CONCLUSION

Among Flemish palliative care physicians there is huge variation regarding religious or ideological views, ranging from traditional Catholics to confirmed atheists. Due to the variety in answers it is difficult to divide the physicians in clearly delineated groups. Like the Belgian general public, many Flemish palliative care physicians concoct their own religious or ideological identity and feel free to drift away from traditional religious and ideological authorities. Nevertheless, our research has shown that among Flemish palliative care physicians, religion is still a factor to be reckoned with.

We contacted all Flemish palliative care physicians, and obtained a response rate of 67.3%, which is relatively high for a sample of physicians, especially when the subject and the length of the questionnaire is taken into account. Therefore, our results are likely to be representative for all palliative care physicians in Flanders. Yet, we did not perform a non-response study to verify the representativeness of the results. We also have been unable to compare our findings with the religious and ideological views and practices of palliative care physicians in other European countries or regions due to a lack of available comparable international data.

Our study clearly showed that attitudes toward euthanasia are influenced by religion and worldview. Though we would never argue that religion and worldview are the only determining factor, they do have an important impact on the way palliative care physicians see a number of delicate ethical issues at the end of life.

## Appendix: The Questionnaire

The respondents were requested to answer the following questions regarding religion and world view.

1. **This question deals with your personal view on religion and world view. Which term best describes your own conviction?**
  Preferably indicate one and in any case maximum two answers.
  □ Christian
  □ Catholic
  □ Protestant
  □ Evangelical
  □ Other Christian denomination (specify): ………………………
  □ Jewish
  □ Muslim
  □ Buddhist
  □ Atheist (I do not believe in God or gods)
  □ Agnostic (Several things are possible)
  □ Free-thinker
  □ New Age
  □ Other world view or religion (specify): ………………………
  □ None of the aforementioned possibilities, because I myself determine what I believe
  □ I am indifferent toward world view and religion
2. **How interesting, important or likely are for you the following aspects of experiencing religion or spirituality? Mark the box that corresponds to your opinion.**
  **Table:**
  		*Not at all*	*A little*	*Average*	*Rather much*	*Very much*
  1.	To what extent are you interested in coming to know more about religion, spirituality, and/or search for the meaning of life?	□	□	□	□	□
  2.	How important are personal prayer and/or meditation to you?	□	□	□	□	□
  3.	In your opinion, how likely is the possibility that God or other transcendentpowers really exist and are not mere human ideas?	□	□	□	□	□
  4.	How important is the attendance of religious or ideological services or ritualsto you?	□	□	□	□	□
3. **Indicate for the following question how often the described situation occurs to you.**
  **Table:**
  		*Never*	*Seldom*	*Some-times*	*Often*	*Often*
  1.	How often do you reflect upon questions relating to religion, spirituality, and/or search for the meaning of life?	□	□	□	□	□
  2.	How often do you encounter situations in which you feel…					
  	a. you are experiencing the presence of God or another transcending power?	□	□	□	□	□
  	b. God or a transcending power wants to say something to you?.	□	□	□	□	□
  	c. God or another transcending force directly intervenes in your life and, in this way, directly determines what happens to you?	□	□	□	□	□
  3.	How often are you actively involved in the planning, organisation or supervision of activities organised by your religious or ideological community?.	□	□	□	□	□
4. **Do you consider yourself a member of a religious or ideological community? Indicate the group to which you belong.**
  Multiple answers are possible.
  □ Catholic Church
  □ Protestant Church (United Protestant Churches of Belgium)
  □ Evangelical Church
  □ Other Christian community (specify): ………………………
  □ Jewish community
  □ Islamic community
  □ Buddhist community
  □ Free-thinking community
  □ Other (specify): ………………………
  □ I do not belong to any group
5. **Besides rituals of transition like weddings, burials and baptisms, how often do you attend religious or ideological rituals (e.g. celebration of the Eucharist)?**
  **Table:**
  	*1. When you were 10 years old*	*2. Now*
  At least once a week	□	□
  At least once a month	□	□
  Several times a year	□	□
  On big feast days	□	□
  Hardly ever or never	□	□
  **What kind of celebrations or rituals are this?**
  1. you were 10 years old (*Skip this question if you hardly or never attended suchcelebrations at that time.*)
    □ Catholic
    □ Protestant
    □ Evangelical
    □ Other Christian (specify): ………………………
    □ Jewish
    □ Islamic
    □ Buddhist
    □ Freemasons
    □ Other (specify): ………………………
  2. Now (*Skip this question if you hardly or never attend such celebrations or rituals.*)
    □ Catholic
    □ Protestant
    □ Evangelical
    □ Other Christian (specify): ………………………
    □ Jewish
    □ Islamic
    □ Buddhist
    □ Freemasons
    □ Other (specify): ………………………
6. **How much importance do you attach to a religious or ideological ritual at the following moments of life? Mark the box that corresponds to your opinion.**
  **Table:**
  		*Entirely unimportant*	*Unimportant*	*Neither important, norunimportant*	*Important*	*Very important*
  1.	Birth	□	□	□	□	□
  2.	Marriage	□	□	□	□	□
  3.	Death	□	□	□	□	□
7. **Besides religious services or rituals, how often do you pray or meditate?**
  □ Daily
  □ At least once a week
  □ At least once a month
  □ Several times a year
  □ Once a year or less
  □ Never
8. **Indicate which view of reality corresponds most closely to your personal conviction.**
  Mark only one answer.
  □ I do not believe there is something transcending human life and the world.
  □ I do not know whether there is something transcending human life and the world.
  □ I believe there is something transcending human life and the world, but I do not know what to visualize with that.
  □ I believe man and world are ruled by a transcendent cosmic principle (e.g. the law of karma).
  □ I believe that also different kinds of trans-human or supernatural entities (ghosts, powers,…) are part of reality.
  □ For me, God is a power of life permeating everything.
  □ I believe in a personal God.
  □ Other (specify): ……………………………
9. **Indicate your opinion about the following statements:**
  **Table:**
  		*Strongly disagree*	*Disagree*	*Neither agree/nor disagree*	*Agree*	*Strongly agree*
  1.	God or (an)other transcending power(s) intervene in our individual lives and solargely determine with whom (partner, friends,…) or what (illness, happiness, accident,…) we will meet…………	□	□	□	□	□
  2.	Finally, God or another transcending power determines when a person dies………………	□	□	□	□	□
10. **Indicate which view about life after death corresponds most closely to your personal conviction.**
  Mark only one answer.
  □ Human beings live on in their work, children,… Yet, I do not believe in life after death (afterlife, reincarnation,…).
  □ I do not know whether there is life after death.
  □ I do believe in life after death, although I have no idea what it may be like.
  □ I believe in a hereafter (heaven).
  □ I believe in heaven and hell.
  □ I believe in reincarnation.
  □ Other (specify): …………………………………
11. **Indicate your personal opinion about the following statement.**
  **Table:**
  		*Strongly disagree*	*Disagree*	*Neither agree/nor disagree*	*Agree*	*Strongly agree*
  After death in an afterlife or in a new life every human will experience the consequences of his actions…………………………………	□	□	□	□	□
12. **Indicate on the scale how important are your personal religion or world view to you within the context of…**
  **Table:**
  		*Entirely unimportant*	*Unimportant*	*Neither important, nor unimportant*	*Important*	*Very important*
  1.	…your life…………………	□	□	□	□	□
  2.	…your professional activities………	□	□	□	□	□
  3.	…your personal attitude toward ethical decisions at the end of life…	□	□	□	□	□
